# Apoptosis regulators of the Bcl‐2 family play a key role in chemoresistance of cholangiocarcinoma organoids

**DOI:** 10.1002/ijc.35483

**Published:** 2025-05-23

**Authors:** Wunan Mi, Gilles S. van Tienderen, Shaojun Shi, Amy Broeders, Kathryn Monfils, Henk P. Roest, Luc J. W. van der Laan, Monique M. A. Verstegen

**Affiliations:** ^1^ Department of Surgery Erasmus MC Transplant Institute, University Medical Center Rotterdam The Netherlands; ^2^ Department of Organ Transplantation Guangdong Provincial People's Hospital (Guangdong Academy of Medical Sciences), Southern Medical University Guangzhou China

**Keywords:** Bcl‐2, chemoresistance, cholangiocarcinoma, gemcitabine, organoids

## Abstract

Cholangiocarcinoma (CCA) is a rare but devastating liver cancer which is commonly diagnosed at a late stage and often resistant to chemotherapy. Bcl‐2 family members, which control apoptotic cell death, are known to be involved in the chemoresistance of some cancer types. This study investigated the role of Bcl‐2 family members in the chemoresistance of cholangiocarcinoma organoids (CCAOs) in both undifferentiated and matured branching phenotypes (BRCCAOs). Patient‐derived CCAOs and BRCCAOs were cultured to assess chemoresistance to an FDA‐approved anticancer drug panel by testing cell viability using ATP quantification and apoptotic cell death by cleaved caspase 3 staining. More specifically, sensitivity to the first‐line drug gemcitabine was tested in combination with Bcl‐2 family inhibitors or activators. We found that in gemcitabine‐resistant CCAOs, inhibition of Bcl‐xl could overcome gemcitabine resistance and induce apoptotic cell death. Although inhibition of Mcl‐1 or activation of Bax induced spontaneous cell death, this could not overcome gemcitabine resistance. The BRCCAOs, which mimic tumor architecture better than CCAOs, show broader chemoresistance to anticancer drugs. Of note, in the resistant BRCCAOs, Bcl‐xl inhibition could restore gemcitabine sensitivity. In conclusion, this study shows that targeting Bcl‐xl can overcome chemoresistance to gemcitabine in CCA organoids. CCAOs and BRCCAOs provide good preclinical models for testing new drug combinations and assessing personalized therapeutic approaches.

## What's New?

Poor prognosis in cholangiocarcinoma (CCA) is attributed to late diagnosis and intrinsic chemoresistance. In particular, resistance mechanisms that suppress apoptosis limit the effectiveness of gemcitabine‐based regimens, which remain the standard treatment. Here, the authors investigated the role of Bcl‐2 family members, which help regulate apoptosis, in CCA chemoresistance. Bcl‐xl was identified as a key driver of gemcitabine resistance in undifferentiated and mature branching phenotypes of patient‐derived CCA organoids. Bcl‐xl inhibition restored gemcitabine‐induced apoptosis, significantly reducing tumor cell viability, even in highly resistant models. The findings highlight Bcl‐xl as a promising target to enhance chemotherapeutic efficacy in CCA.

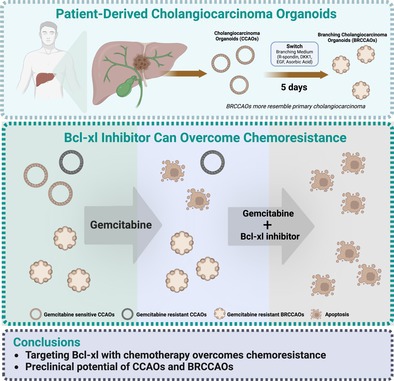

**Abbreviations**
BME: basement membrane extract
BRCCAO: branching cholangiocarcinoma organoid
BSA: bovine serum albumin
CCA: cholangiocarcinoma
CCAO: cholangiocarcinoma organoid
FDA: Food and Drug Administration
H&E: hematoxylin and eosin
IF: immunofluorescence
KRT19: keratin 19
PBS: phosphate buffered saline
PFA: paraformaldehyde
PI: propidium iodide

## 1 INTRODUCTION

Cholangiocarcinoma (CCA) is a rare cancer that arises from biliary epithelial cells, representing the second most prevalent type of primary liver cancer.^1^ Due to a late onset of symptoms, CCA is often diagnosed at advanced stages of disease, missing opportunities for early interventions such as potentially curative surgical resection.^2^ The primary chemotherapy treatment for CCA typically consists of gemcitabine‐based regimens.^3^ Nevertheless, the median survival with single‐agent gemcitabine is only 8 months. Even when combined with cisplatin, the median overall survival in metastatic CCA approaches merely 1 year.^4^ Mostly, this poor survival is attributed to mechanisms of chemoresistance.^5^ There is a clear need for uncovering the molecular basis of chemoresistance. This will aid in designing new, more effective (combination) therapies in CCA treatment.

One of the general hallmarks of cancer is the resistance of tumor cells to apoptosis, which contributes to tumor development and progression.^6^ The Bcl‐2 (B‐cell lymphoma/leukemia‐2 gene) protein family, known for its pivotal role in the regulation of apoptosis, comprises pro‐survival proteins (such as Bcl‐2, Bcl‐xl, Bcl‐w, Mcl‐1, A1/BFL‐1), pro‐apoptotic BH3‐only proteins (including BIM, BID, Puma, Bmf, Noxa, Bik, Bad, Hrk), and effector proteins (Bax, Bak, Bok) responsible for pore‐forming or ‘executioner’ mechanisms.^7^, ^8^, ^9^ Importantly, the responsiveness of malignant tumor cells to apoptosis can be enhanced through either diminishing Bcl‐2 protein expression or suppressing Bcl‐2 functionality.^10^ Bcl‐2 family members are implicated in the chemoresistance of various cancer types. In breast cancer, overexpression of Bcl‐2 has been associated with resistance to anthracyclines and taxanes, potentially due to its role in inhibiting apoptosis and promoting cell survival.^11^ Similarly, in small‐cell lung cancer, Bcl‐xl upregulation contributes to resistance against etoposide and other standard chemotherapeutics, suggesting a potential benefit in targeting Bcl‐xl to sensitize tumor cells to treatment.^1^ Moreover, the cooperative function of Bcl‐2 and Mcl‐1 has been identified as a mechanism leading to treatment failure in acute myeloid leukemia (AML), where dual inhibition of these proteins has shown potential to improve treatment results.^12^ This evidence underscores the therapeutic potential of targeting Bcl‐2 family members to overcome drug resistance mechanisms in cancer treatment.

Several small molecule inhibitors or activators known to target apoptosis‐related Bcl‐2 family proteins have already been employed in clinical trials.^13^, ^14^ For example, ABT‐263 (navitoclax), which inhibits Bcl‐2, Bcl‐xl, and Bcl‐w, was the first BH3‐mimetic drug tested in clinical trials (small‐cell lung cancer and B‐cell malignancies.).^15^ In addition, ABT‐199 (venetoclax) was tested, which is also a BH3‐mimetic drug that highly selectively inhibits Bcl‐2 and therefore effectively induces apoptosis in Bcl‐2 dependent lymphoma.^16^, ^17^ The dependence of Bcl‐xl in cancer cell survival and growth has led to the development of Bcl‐xl‐specific inhibitors such as A‐1155463, A‐1331852, and DT2216, which were shown to potently induce cell death in different cancer types, including colorectal cancer, pancreatic cancer, and leukemia.^18^, ^19^, ^20^ In addition, excessive expression of Mcl‐1 drives tumorigenesis and correlates with poor prognosis, prompting the development and preclinical evaluation of Mcl‐1 inhibitors such as S63845.^21^ However, it remains unclear how these drugs can be effectively used in CCA, especially whether targeting Bcl‐2 family proteins could mitigate gemcitabine resistance, which is the focus of our work.

We have previously described the initiation and expansion of patient derived CCA organoids (CCAOs), capable of sustained expansion for more than 6 months.^22^ These CCAOs broadly maintain the genetic profile of the original tumor and demonstrate tumorigenic potential in xenograft experiments. Additionally, we have also developed branching organoid models (BRCCAOs) that closely mimic CCA tumor tissue at the transcriptional level.^23^ In this study, our aim is to utilize both CCAOs and BRCCAOs models to systematically assess the role of Bcl‐2 family members in gemcitabine chemoresistance. Using this comprehensive approach, we identified Bcl‐xl as a target to enhance the efficacy of gemcitabine treatment and mitigate gemcitabine resistance.

## 2 MATERIALS AND METHODS

### 2.1 Human specimens and organoids culture

Patient‐derived organoid lines (*n* = 3) from CCA were obtained from tumor specimens (approximately 1–4 cm in size, *n* = 3) collected during surgical liver resection at the Erasmus Medical Center Rotterdam. Table S1 summarizes the patient characteristics.

The CCAOs lines were initiated and expanded according to our previously published work.^22^ Briefly, organoid culture involved weekly passage when the organoids reached a visually dense growth state, along with biweekly medium changes. For passaging, organoids were mechanically dissociated by forceful pipetting in ice‐cold Adv DMEM/F12+ (advanced DMEM/F12 supplemented with 100 μg/mL penicillin/streptomycin, Hepes 1 M, 1% Ultraglutamine 200 mM, and Primocin). The cell‐containing solution was diluted with Adv DMEM/F12+ and centrifuged. After removing the supernatant, dissociation and centrifugation steps were repeated. The resulting pellet was suspended in 100% basement membrane extract (BME). The suspension was then plated into cell culture suspension plates, with 20 μL of suspension pipetted into 5 domes per well in a 12‐well plate. Plates were inverted and incubated at 37°C for approximately 30 min before adding expansion medium. BRCCAOs were induced from CCAOs by replacing the expansion medium with branching medium.^23^ Components of expansion medium and branching medium were previously described.^24^

### 2.2 Cell viability assay

Cell viability was assessed using the ATP‐based CellTiter‐Glo 3D Cell Viability Assay (Promega, Madison, WI) according to the manufacturer's instructions. Briefly, the CCAOs were trypsinized into single cells and seeded in a concentration of 1.0 × 10^5^ cells/mL in 96‐well plates (5 μL BME and 100 μL medium per well). Following stimulation with drugs and an equal volume (100 μL) of CellTiter‐Glo reagent was added. Anti‐tumor drugs used in this study are shown in Table S2. The CCAOs were vigorously disrupted for 5 min and then incubated at room temperature for 25 min in the dark. Luminescence was measured using a microplate reader (BMG Labtech, Durham).

### 2.3 FDA‐approved oncology drug screening

Organoids were collected, rinsed with ice‐cold Advanced DMEM, and mechanically fragmented. These fragments were then dissociated into single cells and small cell clusters by subjecting them to three cycles of 3‐min incubations in trypsin–EDTA at 37°C, with mechanical disruption between each cycle. Cells were seeded in 5 μL droplets in white‐walled 96‐well plates, then covered with expansion medium. The oncology drug library (version 8), approved by the Food and Drug Administration (FDA) and provided by the Developmental Therapeutics Program within the Division of Cancer, was tested against a panel of FDA‐approved anticancer drugs (AOD X panel from the NIH National Cancer Institute, *n* = 166 drugs, dtp.cancer.gov). Compounds were introduced at 10 μM concentrations; after 3 days of drug exposure, ATP consent was tested.

### 2.4 Hematoxylin and eosin staining

Organoids were fixed with 4% paraformaldehyde for 20 min, while primary CCA tissue was fixed overnight. The fixed tissue was then dehydrated, embedded in paraffin, and sectioned using standard procedures. Following sectioning, hematoxylin and eosin (H&E) staining was carried out. Images of the H&E stained slides were acquired using a Zeiss Axiokop 20 microscope equipped with a Nikon DS‐U1 camera.

### 2.5 Immunostaining and imaging

For the whole‐mount immunostaining procedure, the organoids underwent an initial wash with phosphate‐buffered saline (PBS) followed by fixation in 4% paraformaldehyde (PFA) for 20 min. Fixed samples were permeabilized with 0.5% Triton X‐100 diluted in 1× PBS for 30 min. Subsequently, samples were blocked with a solution containing 10% serum diluted in 1× PBS for 1 h. Then primary antibodies, diluted in 1% BSA‐1× PBS, were added to the organoids and allowed to incubate overnight at 4°C. Following this, incubation with the secondary antibody (1:500 Invitrogen), also diluted in 1% BSA‐1× PBS, was conducted for 60 min at room temperature in the absence of light. In select cases, additional staining for cytoskeletal components was performed using Phalloidin Alexa Fluor™ 488 (1:200, ThermoFisher) for 60 min at room temperature. Nuclear staining with DAPI (Vector Laboratories) was carried out for 10 min at room temperature. Finally, the samples were imaged using confocal microscopy. In Table S3, all antibody details, including the concentrations used, are listed.

Paraffin‐embedded liver tissues were sectioned (4 μm) and processed for immunofluorescent analysis. For this, sections were deparaffinized and rehydrated through xylene and subsequent ethanol gradient washes according to standard procedures. Antigen retrieval was achieved by heating the sections at 100°C in 10 mM citrate acid buffer (pH 6.0). To minimize nonspecific staining, sections were blocked in a solution containing 1% bovine serum albumin (BSA) and 10% normal goat serum (both from Sigma–Aldrich) in phosphate‐buffered saline (PBS). Subsequently, the same method as whole‐mount immunostaining was employed for incubation with primary antibodies, secondary antibodies, and, specifically, confocal microscopy was used to image the samples.

Brightfield images of the organoids were acquired utilizing the EVOS FL Cell Imaging System (ThermoFisher). For fluorescently stained samples, imaging was performed utilizing the Leica SP5 Intravital confocal microscope, incorporating the APO water dipping lens (40× magnification, 1.00 NA). The lasers operating at 405, 488, and 561 nm were utilized, with emission spectra set at 415–480 nm, 500–550 nm, and 570–700 nm, respectively. Post‐imaging processing was conducted using FIJI (Image J 1.51c, National Institutes of Health, Maryland).

### 2.6 Live/dead immunofluorescent imaging

The staining mixture for live/dead assessment included 12.5 μg/mL propidium iodide (PI, Sigma–Aldrich), 10 μg/mL Hoechst 33342 (Thermo Fisher Scientific), and 0.5 μM Calcein AM (Thermo Fisher Scientific). After staining for 1 h, organoids were imaged using the EVOS FL Cell Imaging System, followed by post‐imaging processing using FIJI.

### 2.7 Western blot assay

The cell suspension was diluted with Adv DMEM/F12+ and centrifuged. After removing the supernatant, organoids were resuspended by forceful pipetting in ice‐cold PBS to remove the basement matrix. The supernatant was removed and the cells were lysed using prepared lysates in ice‐cold buffer containing 2 × Laemmli sample buffer (4% SDS, 20% glycerol, 0.004% bromophenol blue, 0.15 M Tris–HCl, pH 6.8) supplemented with 0.1 mol/L DTT. Subsequently, the cell lysates were heated at 95°C for 10 min. Equal amounts of protein from each sample were loaded onto 10% SDS‐PAGE gels and subsequently transferred onto Immobilon‐PVDF membranes (Millipore) via electroblotting. The proteins in the membranes were then blocked using Odyssey blocking buffer (cat. #927‐70001, Licor‐Biosciences) and incubated overnight at 4°C with primary antibodies. After washing in PBS‐0.05% Tween, the membranes were incubated with secondary antibodies at room temperature for 1 h. The secondary antibodies used were IRDye 680 Goat anti‐Mouse (1:10,000, cat. #926‐68070, Licor‐Biosciences, RRID:AB_10956588) or IRDye 800 Goat anti‐Rabbit (1:10,000, cat. #926‐32211, Licor‐Biosciences, RRID:AB_621843). Protein band intensities were analysed using Image Studio Version 5.5.4 software.

### 2.8 Bulk RNA‐sequencing data

Bulk RNA‐seq data from CCA organoids (CCAOs) was obtained from a previously published study (accession number GSE84073).^22^

### 2.9 Statistical analysis

Statistical analyses were conducted utilizing GraphPad Prism software (v. 9.5.1). Mean values are presented with standard error of the mean (SEM). For comparisons between two matched groups, a two‐tailed paired *t*‐test was employed, while comparisons between two unmatched groups were assessed using a two‐tailed unpaired t‐test. Statistical significance was determined using Kruskal–Wallis's test followed by Dunn's post hoc test (*p < .05, **p < .01, ***p < .001, ****p < .0001).

## 3 RESULTS

### 3.1 Different CCAOs show distinct morphologies and chemo‐sensitivity profiles

As previously published, CCAOs were generated and cultured as a model to detect the response to chemotherapy drugs in CCA (Figure S1A,B).^22^ CCAOs showed morphological differences revealed by microscopic observations and immunofluorescent staining with DAPI (DNA) and phalloidin (actin filaments) (Figure S1C). CCAO1 shows organoids with a regular spherical structure with a hollow interior and thin walls, with few necrotic cell fragments in the center of the organoids. CCAO2 exhibits an irregular, compact morphology with thicker multicyclic walls. CCAO3 resembles CCAO1 but lacks detached cells at the center of the organoids. This variability may be attributed to genetic variations among patients, which may also result in differences in sensitivity to various chemotherapeutic drugs.

**FIGURE 1:**
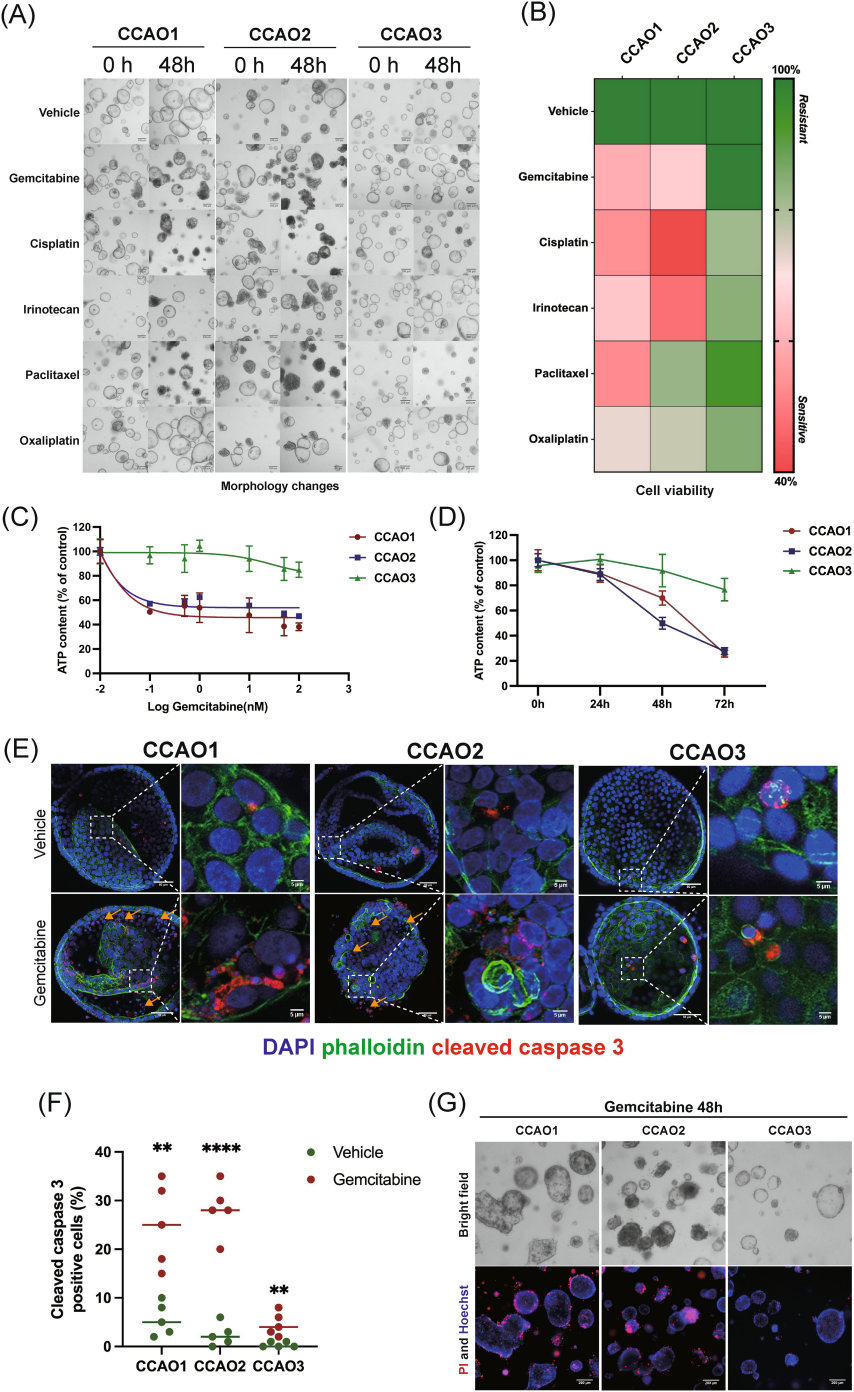
Susceptibility and resistance to gemcitabine in CCAOs. (A) Phenotypic changes of the CCAOs were visualized using brightfield microscopy after 48 h of exposure to drugs. (B) Cell viability was expressed as a heatmap in which red represents sensitivity to the drug, expressed as a decrease in ATP content, while green represents resistance. (C) CCAOs (*n* = 3) were cultured with increasing doses of gemcitabine (ranging from 0 to 100 μM) for 48 h, followed by assessment of cell viability using the CellTiter Glo assay. (D) CCAOs were treated with 1 μM Gemcitabine for 24, 48, and 72 h, after which cell viability was evaluated using an ATP content assay. (E) and (F) CCAOs (*n* = 3) were subjected to gemcitabine (1 μM) for 48 h, followed by immunofluorescent staining for cleaved caspase 3 (red) to assess cell death. Representative fluorescent images were captured, and quantification of positive cells in the images was conducted. (G) Brightfield and PI/Hoechst double staining were performed on CCAOs after 48 h of stimulation with gemcitabine (1 μM). Shown is one representative experiment of three (*p < .05, **p < .01, ***p < .001, ****p < .0001).

We employed two evaluation methods (morphological changes and cell viability) to determine the sensitivity to commonly used chemotherapeutic agents in the three CCAO lines (Figure S1D). Following stimulation, a CellTiter‐Glo assay was performed to evaluate ATP content to assess cell viability after 48 h, and brightfield microscopy was utilized to study morphological alterations. Five drugs, commonly used in the clinic, gemcitabine (1 μM), cisplatin (10 μM), irinotecan (10 μM), paclitaxel (1 μM), and oxaliplatin (10 μM) were selected. We first studied morphological changes after 48 h of treatment (Figure 1A). Notably, gemcitabine and cisplatin induced morphological changes in CCAO1 and CCAO2, characterized by a lack of growth and the presence of cellular debris within and around the organoids. Conversely, CCAO3 displayed resistance to most chemotherapeutic drugs, except paclitaxel, with almost no alteration in morphology and even continued growth (addressed as increase in organoid size). Correspondingly, we assessed the viability of cells post 48‐h drug treatment and summarized the results in a heatmap (Figure 1B); in contrast to CCAO1 and CCAO2, CCAO3 demonstrated to be insensitive to all chemotherapeutic agents tested, particularly to the frontline chemotherapeutic agent gemcitabine.

### 3.2 Gemcitabine susceptibility and resistance in CCAOs

To further elucidate the resistance of CCAO3 to gemcitabine treatment, we exposed CCAOs to increasing concentrations of gemcitabine (0.1–100 μM). As expected, even at a low concentration of gemcitabine (0.1 μM) after 48 h of stimulation, the cell viability of CCAO1 and 2 decreased to 50% and 57%, while CCAO3 remained (97%) (Figure 1C). Moreover, at each concentration (0.1, 0.5, 1, 10, 50, and 100 μM) of gemcitabine, CCAO3 showed significant resistance (*p* < .05) compared to CCAO1 and 2. In addition, we used 1 μM gemcitabine to treat the CCAOs and analyze cell viability at different time intervals (24, 48, and 72 h) (Figure 1D). The results showed that from 0 to 72 h, the relative cell viability of CCAO1 and 2 dropped to 26% and 28%, respectively. The relative cell viability of CCAO3 only decreased to 77%.

To further compare the induction of programmed cell death pathways by gemcitabine in CCAOs, immunofluorescence was conducted to detect the apoptotic marker cleaved caspase 3. Following gemcitabine (1 μM) treatment for 48 h, approximately 20–30% of cells in CCAO1 and CCAO2 expressed cleaved caspase 3 (Figure 1E,F). Moreover, there was evident nuclear condensation, presenting an overall smaller and denser morphology. These fragmented cells detached from the entire organoid framework, losing their intact cellular structure. In contrast, CCAO3 exhibited intact morphology with no evidence of nuclear condensation or cellular fragmentation. The expression of the apoptotic marker cleaved caspase 3 increased by only about 5% compared to the vehicle group. PI and Hoechst staining results demonstrated that the number of dead cells in CCAO3 was lower than in CCAO1 and CCAO2 (Figure 1G). The presented results support the relative resistance of CCAO3 to gemcitabine.

### 3.3 Specific role of Bcl‐2 family member Bcl‐xl in gemcitabine‐resistant CCAOs

**FIGURE 2:**
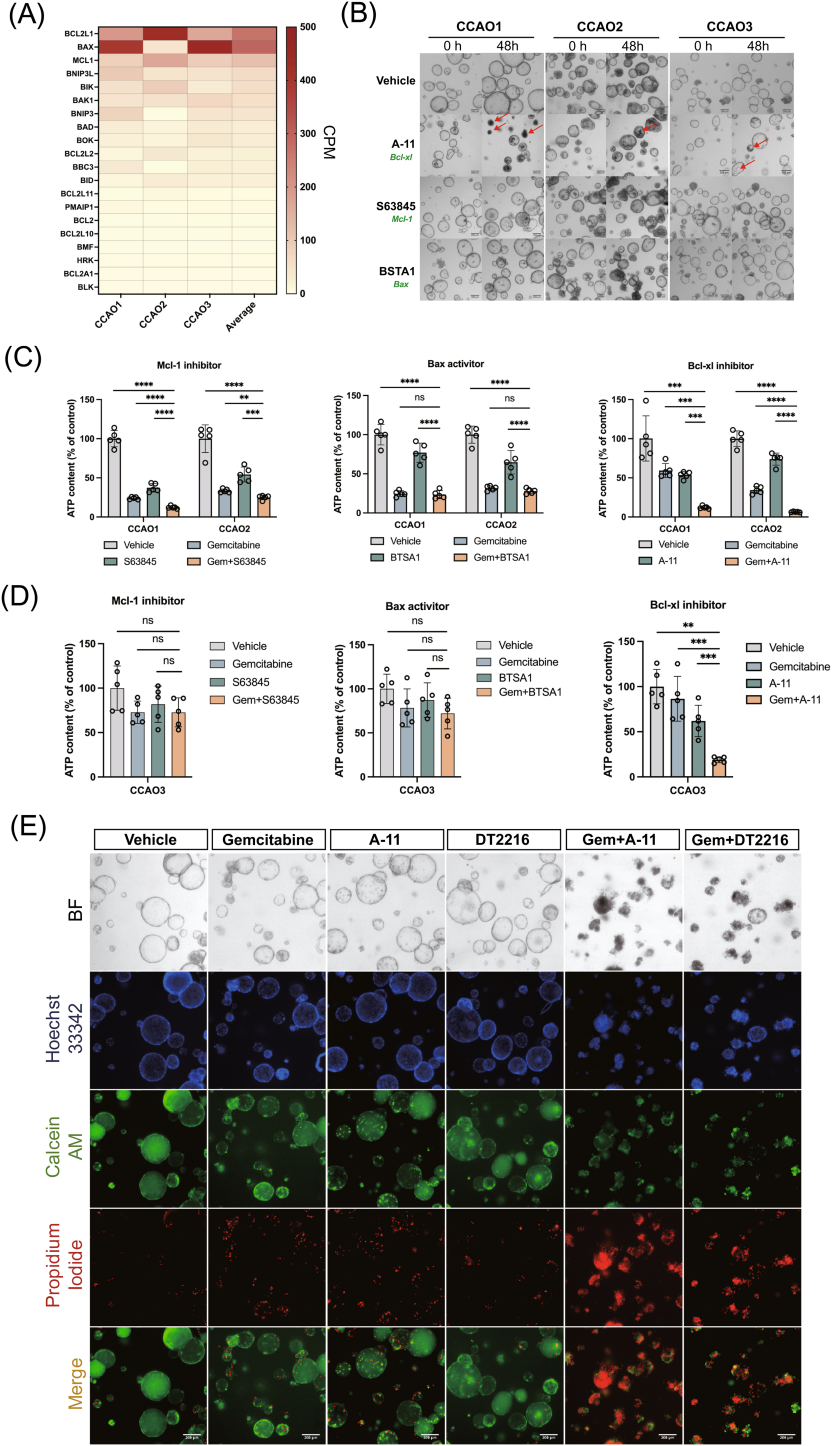
Specific role of Bcl‐2 family member Bcl‐xl in gemcitabine resistance. (A) Heatmap showing the relative abundance of gene expression for Bcl‐2 family members in CCAOs (*n* = 3) as determined by RNA sequencing. Gene expression values are represented in counts per million (CPM), a normalization method that adjusts for sequencing depth across samples. (B) CCAOs were exposed to inhibitors of Bcl‐2 family members A‐11 (5 μM) and S63845 (10 μM) or Bax activator BSTA1 (10 μM) for 48 h. Shown are representative brightfield images from three independent experiments. (C, D) Co‐targeting gemcitabine with Bcl‐2 family inhibitors or activator in gemcitabine sensitive (CCAO1 and CCAO2) or resistant (CCAO3) organoids lines. Cell viability was assessed by measuring ATP content after 48 h treatment with gemcitabine (1 μM) in combination with inhibitors A‐11 (5 μM), S63845 (10 μM) or activator BSTA1 (10 μM). (E) Representative brightfield and immunofluorescent images of life‐dead staining using Calcein (live, green), PI (dead, red) and Hoechst (nuclei, blue) for CCAO3. CCAO3 was exposed for 48 h to 1 μM gemcitabine, 5 μM A‐11, or 10 μM DT2216, and combinations of these (*p < .05, **p < .01, ***p < .001, ****p < .0001).

To explore strategies for overcoming gemcitabine resistance, we further focused on the apoptosis‐related Bcl‐2 family. Initially, mRNA sequencing of CCAO1, CCAO2, and CCAO3 was implemented, and the expression levels of members of the Bcl‐2 family associated with apoptosis were summarized in a heatmap (Figure 2A). Compared to other Bcl2 family members, we found relatively high average mRNA levels of Bcl‐xl (BCL2L1), Bax, and Mcl‐1 in the three tumor organoid lines, while another key member Bcl‐2 exhibited comparatively lower expression. Next, small molecule inhibitors or activators targeting Bcl‐xl (A‐1155463 5 μM) (A‐11), Mcl‐1(S63854 10 μM), and Bax (BSTA1 10 μM) were applied to CCAOs to assess the effects on cell viability and spontaneous cell death. As shown in Figure 2B, a 48‐h treatment with S63854 and BSTA1 resulted in only modest morphological changes across all three CCAOs. In contrast, the Bcl‐xl inhibitor A‐11 induced pronounced morphological alterations within 48 h (indicated by red arrows), with the most significant effects observed in CCAO1.

**FIGURE 3:**
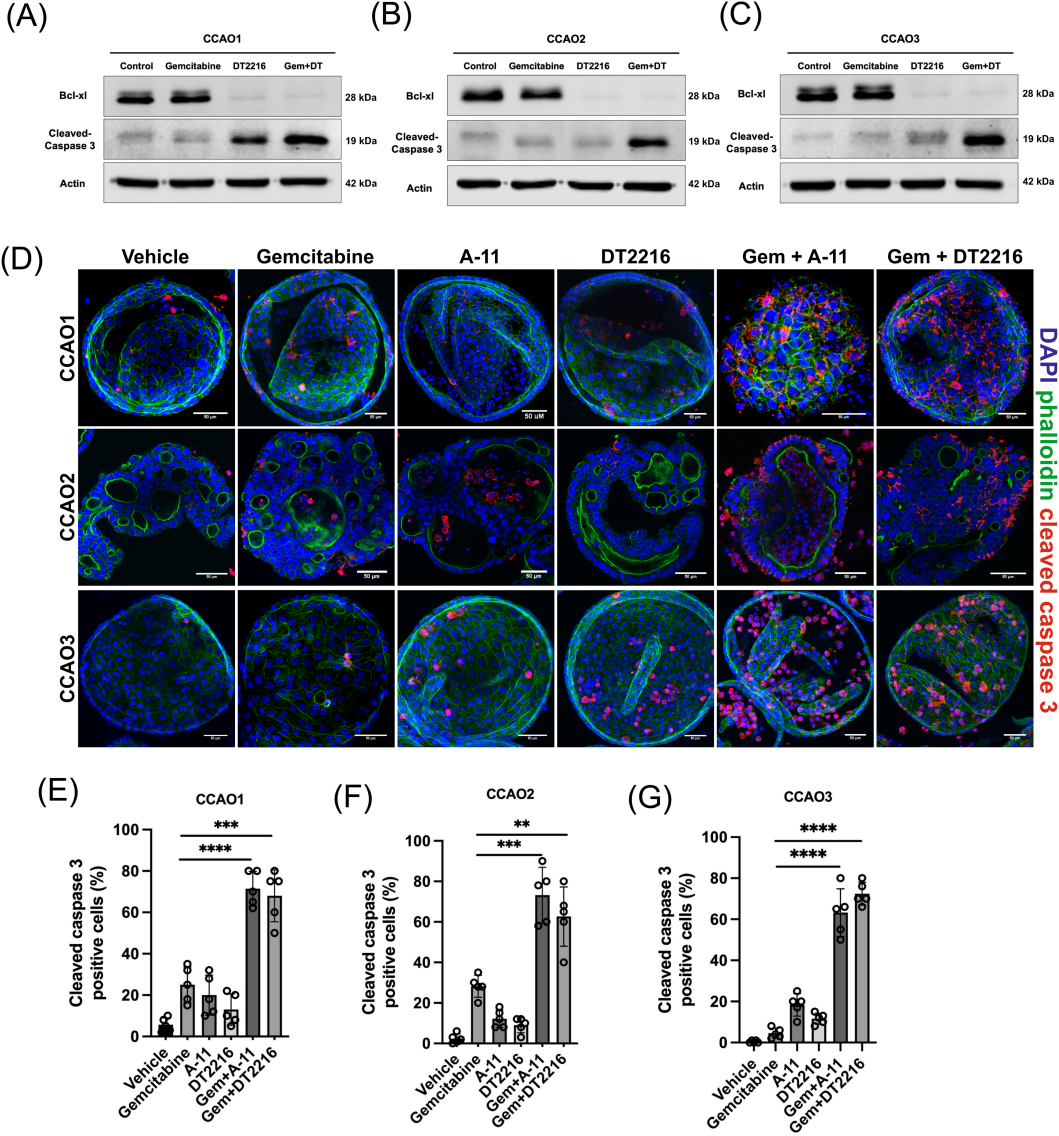
Bcl‐xl inhibition overcomes resistance to gemcitabine‐induced cell death. (A–C) Bcl‐xl, Cleaved‐caspase 3, and Actin proteins were detected by western blotting. CCAOs were treated with 1 μM gemcitabine, 10 μM DT2216, or their combination for 24 h. (D) Cleaved caspase 3 expression in CCAOs were analysed after incubation with gemcitabine 1 μM, A‐11 5 μM, DT2216 10 μM, or combinations. Cleaved caspase 3 (red), nuclei (blue) and phalloidin (green) are shown merged. (E–G) Positive cell quantification from images was performed and showed significant differences in apoptotic cell populations among the different treatments, as depicted in the statistical charts (*p < .05, **p < .01, ***p < .001, ****p < .0001).

To assess the combined effects of Bcl‐2 family members with gemcitabine, we co‐administered gemcitabine with A‐11, S63854, or BSTA1 and evaluated changes in viable cells by ATP quantification. The results revealed that the combination of gemcitabine with the Bcl‐xl inhibitor A‐11 showed an enhanced cytotoxic effect in the gemcitabine‐sensitive organoids CCAO1 and CCAO2 (*p* < .001; Figure 2C). In the gemcitabine‐sensitive organoids, the Mcl‐1 inhibitor S64845 also significantly enhanced both spontaneous and gemcitabine‐induced cytotoxicity (*p* < .001, Figure 2C). The Bax activator BSTA1 induced spontaneous cytotoxicity but had no significant effect on the gemcitabine‐induced cytotoxicity (Figure 2C). When looking at gemcitabine‐resistant organoid lines, CCAO3, only the Bcl‐xl inhibitor (*p* < .001) and not the Mcl‐1 inhibitor or Bax activator could overcome gemcitabine resistance as assessed by ATP content measurements (Figure 2D). To confirm the role of Bcl‐xl, we tested a second small molecule inhibitor, DT2216 (10 μM). As shown in Figure S2, both A‐11 and DT2216 gave similar results in both gemcitabine‐sensitive CCAOs and the gemcitabine‐resistant CCAO3. This finding confirms that the effects of both inhibitors are likely to be specific for Bcl‐xl. To confirm that gemcitabine induces cell death in CCAOs, we conducted Calcein AM and PI staining. As shown in Figure 2E, the combination of gemcitabine and Bcl‐xl inhibitors increased cell death in CCAO3, effectively overcoming gemcitabine resistance in these organoids. Finally, we assessed the effect of Bcl‐xl inhibitors A‐11 and DT2216 on cisplatin‐treated organoids. As shown in Figure S3, cisplatin induced cytotoxic effects in both CCAO1 and CCAO2 and had only a limited effect in CCAO3. Combining A‐11 or DT2216 with cisplatin significantly enhanced the cytotoxic effects even in the cisplatin‐resistant CCAO3 (Figure S3A,B, *p* < .0001). To test the effect of the Bcl‐xl inhibitor DT2216 on Bcl‐xl protein levels in CCAOs, we performed Western blot analyses. As shown in Figure 3A–C, DT2216 treatment effectively induced the degradation of Bcl‐xl protein in all CCAOs, also in combination with gemcitabine, confirming the drug's efficacy.

We further investigated the cell death mechanism induced by gemcitabine. To confirm whether gemcitabine induces apoptosis in CCAOs, we performed western blotting to detect cleaved caspase 3. As shown in Figure 3A–C, gemcitabine or DT2216 treatment alone did not clearly increase the levels of cleaved caspase 3. However, when gemcitabine and DT2216 were combined, a marked increase in cleaved caspase 3 production was evident across all three CCAOs. Similarly, staining results indicated that single usage of gemcitabine or Bcl‐xl inhibitors (A‐11 or DT2216) induced cleaved caspase 3 in only a small fraction of cells, with relatively intact organoid structures (Figure 3D–G). However, after 48 h of combined usage on CCAOs, over 60% of cells exhibited clear apoptotic signals (*p* < .01), and the organoid structures were disrupted, with many cell fragments scattered in and around the organoids. This further supports the notion that the additive mechanism of gemcitabine and Bcl‐xl inhibitors involves inducing more pronounced apoptosis in CCAOs.

### 3.4 BRCCAOs are more sensitive to Bcl‐xl inhibitor A‐11 than CCAOs

**FIGURE 4:**
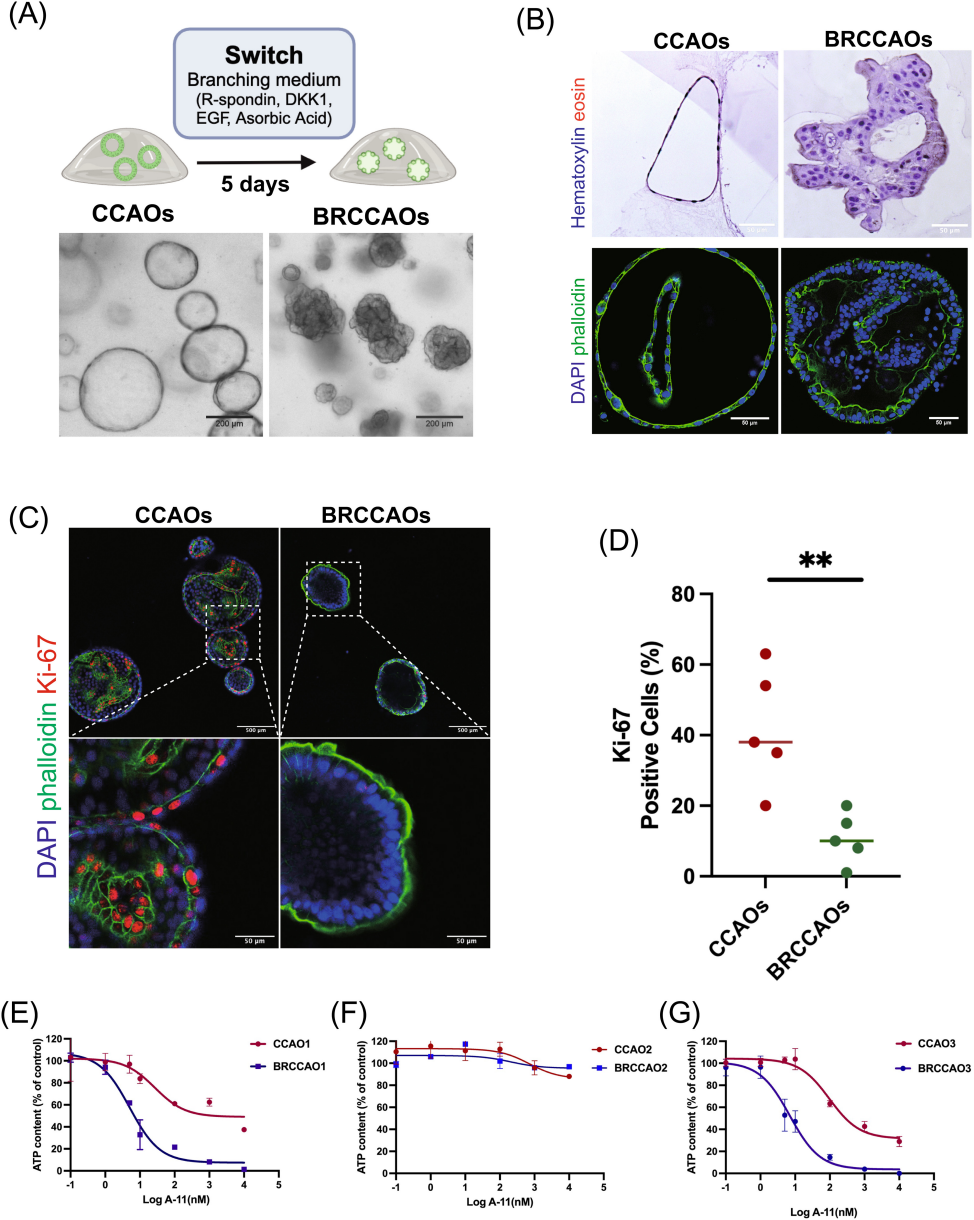
Branching morphogenesis in CCAOs reduces cell proliferation and increases response to Bcl‐xl inhibition. (A, B) Representative images highlight the morphological distinctions between CCAOs and BRCCAOs. (C) Representative Ki‐67 (red) immunofluorescent staining in CCAOs and BRCCAOs. Cells were co‐stained with DAPI for nuclei (blue) and phalloidin (green). (D) Quantification of Ki‐67 positive cells for CCAOs and BRCCAOs showing a significant reduction of cell proliferation in BRCCAOs. (E–G) CCAOs (*n* = 3) and BRCCAOs were cultured with increasing doses of A‐11 (ranging from 0.1 to 10,000 nM) for 48 h, followed by assessment of cell viability using the CellTiter Glo assay (*p < .05, **p < .01, ***p < .001, ****p < .0001).

To confirm the findings in the CCAO lines, we extended the analysis to the branching type, BRCCAO, that has a more matured phenotype and better resembles the CCA tumor (Figure 4A,B).^23^ BRCCAOs expressed Ki67 (10.8%) showing their lower cell proliferation rate compared to CCAOs (*p* < .05) (Figure 4C,D).

Increasing concentrations of A‐11 (0.1, 1, 10, 100, 1000, and 10,000 nM) were applied to the three CCAO and BRCCAO lines for 48 h. Cell viability assessment showed that BRCCAO1 and 3 are more sensitive to A‐11 compared to their CCAO counterparts (Figure 4E–G). With increasing concentration of A‐11, ATP content decreased more rapidly in BRCCAO: at 100 nM, ATP content in CCAO1 dropped to 61%, whereas in BRCCAO1, it dropped to 22%, significantly lower than CCAO1 (*p* < .05). Similarly, in CCAO3, the ATP content decreased to 63% at 100 nM, while in BRCCAO3 a decrease to 15% (*p* < .05) was measured. Interestingly, CCAO2 and BRCCAO2 demonstrated to be more resistant to A‐11. Even at the highest concentration of 10,000 nM, the ATP content in CCAO2 and BRCCAO2 remained at 113% and 103%, respectively.

### 3.5 BRCCAOs demonstrate resistance to gemcitabine

**FIGURE 5:**
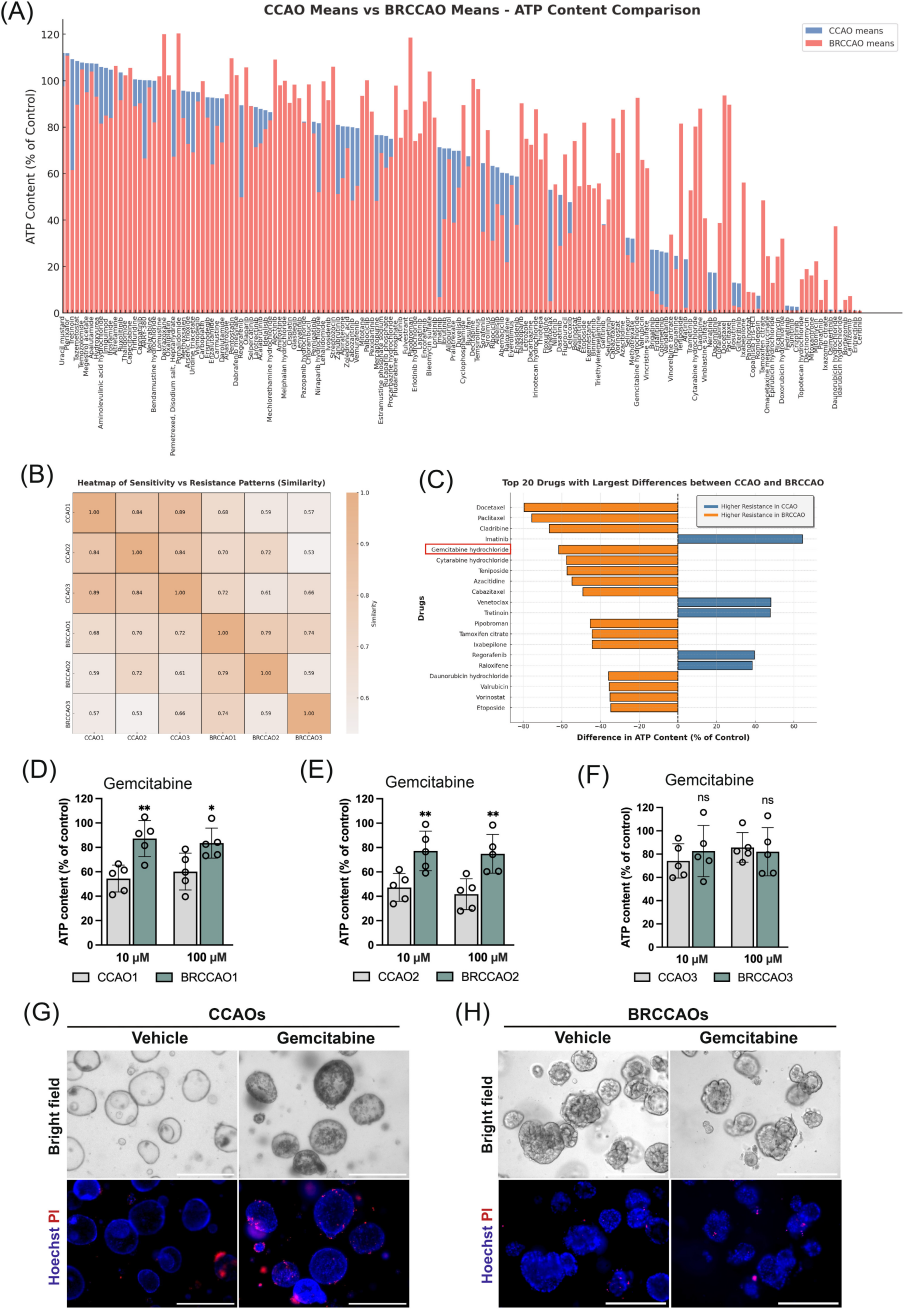
BRCCAOs show broader chemoresistance and are more resistant to gemcitabine. (A) Comparison of ATP content (% of control) between CCAOs and BRCCAOs groups across various chemotherapeutics. The blue bars represent the ATP content for the CCAOs group, while the red bars represent the BRCCAOs group. (B) This heatmap shows the degree of similarity in drug response patterns between three CCAO (CCAO1, CCAO2, CCAO3) and three BRCCAO (BRCCAO1, BRCCAO2, BRCCAO3) groups. The values in each cell represent the correlation coefficients (ranging from 0.5 to 1.0). The color gradient represents the strength of similarity. (C) Top 20 Drugs with Largest Differences between CCAOs and BRCCAOs. The horizontal bar chart represents the differences in ATP content (% of control) between CCAOs and BRCCAOs. (D–F) BRCCAOs were subjected to a 48‐h treatment with 10 or 100 μM gemcitabine, followed by CellTiter Glo assay. (G and H) Representative brightfield and immunofluorescence images of CCAOs and BRCCAOs stained with DAPI and PI (green and red) after 48‐h exposure to 10 μM gemcitabine. Scale bars indicate 300 μm (*p < .05, **p < .01, ***p < .001, ****p < .0001).

The sensitivity of CCAOs and BRCCAOs for FDA‐approved anticancer drugs (AOD X panel from the NIH National Cancer Institute, *n* = 166 drugs) was assessed in a drug screening (Figure 5A). Overall, BRCCAOs exhibited a higher level of resistance compared to CCAOs for the majority of drugs tested, with BRCCAOs being more resistant to 65% of the drugs while demonstrating increased sensitivity to 35%. A heatmap was generated to visualize the responses per drug in both CCAOs and BRCCAOs (Figure 5B). The CCAO models (CCAO1, CCAO2, CCAO3) showed similar responses to the drugs, with correlation values ranging from 0.85 to 0.9. In contrast, the BRCCAO models (BRCCAO1, BRCCAO2, BRCCAO3) showed more heterogeneous response patterns, with correlation values between 0.6 and 0.81, indicating greater variability in drug resistance. Cross‐group comparisons (CCAO vs. BRCCAO) exhibited generally lower correlation values, further highlighting differences in resistance patterns.

As shown in Figure 5C, the top 20 drugs with the largest differences between CCAOs and BRCCAOs are listed. For 14 drugs, a significantly increased drug resistance was observed in the branching organoids. Notably, docetaxel and paclitaxel displayed the largest effect, with BRCCAOs exhibiting up to 80% more ATP content relative to CCAOs. Other notable drugs in this category included cladribine, gemcitabine, and cytarabine, all demonstrating an average increase of over 50% in ATP content in BRCCAOs compared to CCAOs. Conversely, six drugs showed higher resistance in CCAOs. Among these, imatinib, venetoclax, and tretinoin exhibited the largest differences in ATP content, with values ranging from 40% to 60% higher in CCAOs compared to BRCCAOs, suggesting that these drugs may be more effective against the branching differentiation state. The overall pattern suggests that BRCCAOs are generally more resistant to a broader range of drugs, with significantly higher resistance to many standard chemotherapy agents. In contrast, CCAOs demonstrated resistance to fewer drugs but displayed greater sensitivity to some targeted therapies.

To further validate the gemcitabine resistance in BRCCAOs, we exposed both CCAOs and BRCCAOs to 10 and 100 μM of gemcitabine for 48 h, followed by measurement of ATP content (Figure 5D–F). The results indicated that gemcitabine‐sensitive organoids (CCAO1 and CCAO2) become significantly more resistant when matured to a branching phenotype (Figure 5D,E, *p* < .05). For gemcitabine‐resistant organoids CCAO3, no significant increase in response to gemcitabine was observed after maturation to a branching phenotype (Figure 5F). Furthermore, we assessed the number of dead cells by PI staining on CCAOs and BRCCAOs following 48 h of treatment with 10 μM gemcitabine. The results showed that only CCAOs were sensitive to gemcitabine, as indicated by both morphological changes and the number of PI‐positive cells clearly shown in Figure 5G,H. In contrast, BRCCAOs displayed no signs of cell death in response to gemcitabine.

### 3.6 Bcl‐xl contributes to gemcitabine chemoresistance in branching CCAOs

**FIGURE 6:**
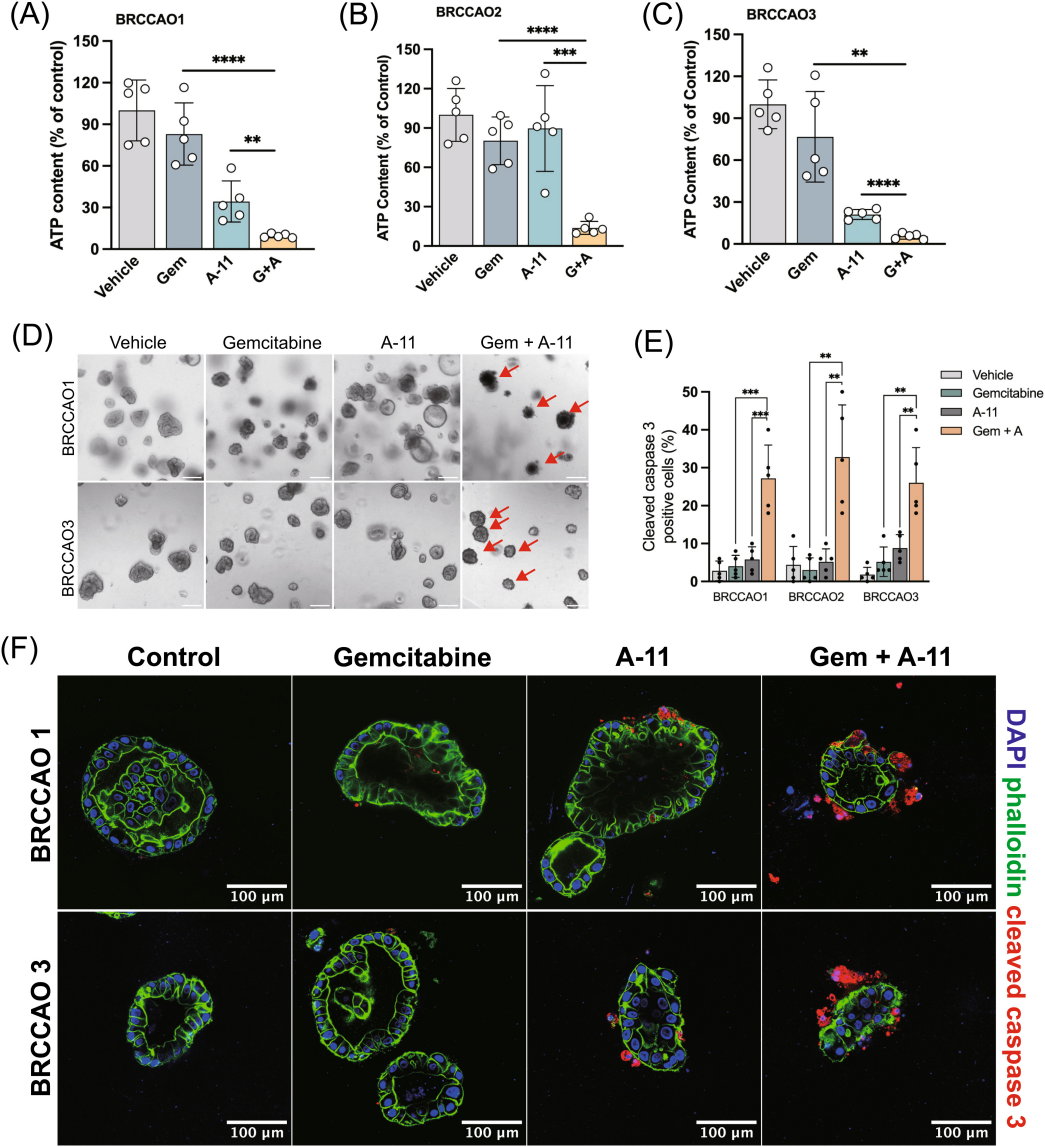
Bcl‐xl inhibitor also overcome gemcitabine‐resistance in BRCCAOs. (A–C) BRCCAOs were subjected to a 48‐h treatment with a combination of 10 μM gemcitabine and 0.2 nM A‐11, followed by cell viability assessment. (D) BRCCAOs were exposed to gemcitabine (10 μM), A‐11 (100 nM), and their combination for 48 h, and subsequent morphological changes were recorded. All scare bars indicate 200 μm. (E, F) BRCCAOs were treated with 10 μM gemcitabine, 100 nM A‐11, or their combination, followed by staining for cleaved caspase 3 (red), phalloidin (green), and DAPI (blue) (*p < .05, **p < .01, ***p < .001, ****p < .0001).

We aimed to explore the enhanced cytotoxic effect of gemcitabine in combination with the Bcl‐xl inhibitor using BRCCAOs to confirm our findings in CCAOs. BRCCAOs were treated for 48 h with 10 μM gemcitabine alone, 100 nM A‐11 alone, or gemcitabine and A‐11 combined. We assessed cell viability changes using the CTG assay (Figure 6). The results for BRCCAO1 (Figure 6A), BRCCAO2 (Figure 6B) and BRCCAO3 (Figure 6C) show that A‐11, combined with gemcitabine, significantly decreased cell viability when compared to either A‐11 or gemcitabine alone (*p* < .0001). With combination treatment, ATP content dropped below 10%. Furthermore, we imaged the morphological changes in BRCCAOs (Figure 6D). The results showed that the combination of gemcitabine with the Bcl‐xl inhibitor A‐11 disrupted the morphology of branching organoids, as indicated by the red arrows. To confirm increased apoptosis, we performed IF staining for the apoptosis marker cleaved caspase 3 (Figure 6E,F). The results showed that, following combination treatment, the proportion of cleaved caspase 3 positive cells increased to 27.2%, 32.8%, and 26% in BRCCAO1, BRCCAO2, and BRCCAO3 (*p* < .05), respectively. Additionally, we observed morphological changes in the organoids, including shrinkage, fragmentation, and numerous cell debris surrounding the organoids. Therefore, we conclude that the combination of gemcitabine and Bcl‐xl inhibitors also exerts additive effects in BRCCAOs, overcoming gemcitabine resistance.

## 4 DISCUSSION

Tumor drug resistance is a critical factor in clinical treatment failures, impacting patient outcomes across cancer patients.^25^ In CCA, resistance to the first‐line chemotherapy agent gemcitabine arises from multiple complex factors such as enhanced drug efflux, alterations in drug targets, increased DNA repair, and notably, evasion of apoptosis.^26^ These resistance mechanisms could be influenced by the Bcl‐2 family of proteins, which regulate the intrinsic or mitochondrial apoptosis pathway.^27^, ^28^ Especially, increased expression of key anti‐apoptotic proteins, such as Bcl‐2, Bcl‐xl, and Mcl‐1, has been proven to inhibit pro‐apoptotic factors and prevent the apoptosis process.^29^, ^30^, ^31^ Additionally, many selective inhibitors have been developed; however, solid tumors normally show resistance to these drugs when used as single‐agent treatment.^32^ In our research, similar outcomes were observed when Bcl‐xl or Mcl‐1 inhibitors, or Bax activator on their own were used in CCAOs, showing limited effectiveness. Therefore, combining these Bcl‐2 family inhibitors with gemcitabine may offer a more effective strategy.

Previous studies show that combining Bcl‐2 family inhibitors such as ABT‐737 with gemcitabine enhances apoptosis in pancreatic cancer cells, overcoming drug resistance mechanisms.^33^ In non‐small cell lung cancer, the dual application of ABT‐263, which also inhibits Bcl‐2, Bcl‐xl, and Bcl‐w, with gemcitabine has shown improved therapeutic outcomes by effectively reducing tumor growth and increasing survival rates in preclinical models.^34^ Notable studies include the use of DT2216, a Bcl‐xl‐specific degrader, which, when combined with gemcitabine, has shown to effectively overcome resistance in pancreatic cancer by promoting apoptosis more efficiently than either agent on its own.^20^ Furthermore, in nasopharyngeal carcinoma, the combination of gemcitabine with APG‐1252, a novel inhibitor of Bcl‐2/Bcl‐xl, exhibited additive antitumor effects. This combination disrupts the Jak‐2/Stat3/Mcl‐1 pathway, highlighting a novel mechanistic approach to potentiate gemcitabine's efficacy.^35^ Also, additive antitumor activity has been observed with gemcitabine and ABT‐737 in various cancers, where their combined effect disrupts the interaction between USP9X and Mcl‐1, enhancing apoptosis and potentially overcoming inherent drug resistance.^36^ Whether the observed combined effects of Bcl‐xl inhibition and gemcitabine in the CCAOs were merely additive or truly synergistic remains to be determined and requires further in‐depth analyses.

Here, we specifically targeted three Bcl‐2 family members: Bcl‐xl, Bax, and Mcl‐1, that showed the highest mRNA expression in CCAOs. Utilizing the respective small molecule inhibitors or activator in combination with gemcitabine, we confirmed that only the combination involving a Bcl‐xl inhibitor significantly overcame gemcitabine resistance, effectively inducing apoptosis. Notably, recent studies across cancer patients underscore the significant role of Bcl‐xl in mediating drug resistance, particularly highlighting its potential as a target for combination therapy. For example, in pancreatic, breast, lung, and gastric cancers, Bcl‐xl has been shown to confer resistance to apoptosis and enhance tumor survival against targeted therapies.^37^, ^38^, ^39^ These findings suggest that targeting Bcl‐xl, especially in combination with agents such as gemcitabine, could significantly improve treatment outcomes by overcoming drug resistance.

Of note, our study involved tumor organoids derived from three different patients, which displayed varying responses to gemcitabine and Bcl‐2 family inhibitors. However, the combined use of gemcitabine and a Bcl‐xl inhibitor consistently demonstrated additive effects across these organoid models. This variability in individual responses further substantiates the reliability of our findings and highlights the importance of personalized approaches in the treatment of cholangiocarcinoma.

Although it is challenging to get a 100% pure BRCCAOs culture, which necessitates handpicking of the branching structures from the cystic organoids, BRCCAOs have been shown to be a better model for CCA than the conventionally grown CCAOs.^23^ Using BRCCAOs in our research has therefore provided profound insights into the cellular mechanisms of gemcitabine resistance in CCA, particularly focusing on the role of Bcl‐xl in this. We have demonstrated that inhibiting Bcl‐xl can restore sensitivity to gemcitabine, suggesting a potential clinical application for Bcl‐xl inhibitors in combination therapy to overcome resistance.

In summary, we demonstrated that Bcl‐xl inhibition restored gemcitabine sensitivity in our most resistant CCA organoid model (CCAO3) and in BRCCAOs. However, since our study was conducted on a limited number of patient‐derived organoid lines, these findings may not apply to all CCA tumors. Future studies incorporating a larger number of CCA lines and co‐culture systems that include stromal and immune components will be necessary to validate our findings and assess the broader therapeutic relevance of Bcl‐xl inhibition in CCA. Additionally, while our study identifies Bcl‐xl as a promising target, we acknowledge that other apoptosis regulators, such as IAPs, may also play critical roles in chemoresistance.^40^ For instance, IAPs have been shown to contribute to gemcitabine resistance in CCA, highlighting the need for further exploration of alternative apoptotic pathways.

## AUTHOR CONTRIBUTIONS

**Wunan Mi:** Conceptualization; software; resources; data curation; formal analysis; investigation; writing – original draft; writing – review and editing. **Gilles S. van Tienderen:** Resources; investigation; methodology. **Shaojun Shi:** Resources; investigation; methodology. **Amy Broeders:** Resources. **Kathryn Monfils:** Resources. **Henk P. Roest:** Resources; data curation. **Luc J. W. van der Laan:** Conceptualization; supervision; funding acquisition; project administration; writing – review and editing. **Monique M. A. Verstegen:** Conceptualization; funding acquisition; writing – review and editing; project administration; supervision.

## CONFLICT OF INTEREST STATEMENT

The authors have no potential competing interests to declare.

## ETHICS STATEMENT

Ethical approval for CCA tissue usage in research was obtained from the Medical Ethical Council of the Erasmus MC, and written informed consent was provided by all patients (MEC‐2013‐143).

## Supporting information


**Appendix S1:** Supporting information.

## Data Availability

The data that support the findings of this study are available from the corresponding author upon reasonable request.
