# Supplementary material for: Apoptosis regulators of the Bcl‐2 family play a key role in chemoresistance of cholangiocarcinoma organoids
Source: Int J Cancer. 2025 May 23;157(8):1694–708. doi: 10.1002/ijc.35483 (PMC12375844; doi:10.1002/ijc.35483)
Supplement: Supplementary file 1 — Appendix S1: Supporting information. [file IJC-157-1694-s001.pdf]

Apoptosis regulators of the Bcl-2 family play a key role in chemoresistance of cholangiocarcinoma organoids

Wunan Mi, Gilles S. van Tienderen, Shaojun Shi, Amy Broeders, Kathryn Monfils, Henk P. Roest, Luc J.W. van der Laan and Monique M.A. Verstegen

Contents

Supplementary tables……………………………………………………………………2

Table S1. The main characteristics of patients.………………………………..….2

Table S2. List of anti-tumor drugs used in this study.……………………..…..….2

Table S3. List of primary antibodies used in this study.…..………………….….2

Supplementary figures……………………….…………………………………………3

Figure S1. Cholangiocarcinoma tumor histology and sample processing..………3

Figure S2. Brightfield images depicting morphological alterations…………..….4

Figure S3. Drug responses to Cisplatin…………………………….……………….5

Supplementary tables

Table S1 The main characteristics of patients

Culture type	Age patient (years)	Sex	Cell Source
CCAO1, BRCCAO1	34	F	pCCA
CCAO2, BRCCAO2	59	M	iCCA
CCAO3, BRCCAO3	59	M	iCCA

Abbreviations. CCAO: cholangiocarcinoma organoid; BRCCAO: Branching cholangiocarcinoma organoid; pCCA: perihilar cholangiocarcinoma; iCCA: intrahepatic cholangiocarcinoma, n/a: not applicable, n/p: not performed.

Table S2 List of anti-tumor drugs used in this study

Drugs	Source	Identifier
Gemcitabine	Accord Healthcare	n/a
Cisplatin	Accord Healthcare	n/a
Irinotecan	Accord Healthcare	n/a
Paclitaxel	Accord Healthcare	n/a
Oxaliplatin	Accord Healthcare	n/a
ABT-199	Merck Chemicals BV	HY-15531
ABT-236	Merck Chemicals BV	HY-10087
S63845	Merck Chemicals BV	HY-100741
BTSA1	Merck Chemicals BV	HY-123054
A-1155463	Merck Chemicals BV	HY-19725
DT2216	Selleck Chemicals	S8924

Table S3 List of primary antibodies used in this study

Antibody	Source	Identifier	Concentration
Active caspase 3 (Cleaved caspase 3)	R&D Systems	AF835	IF: 4ug/ml
Cleaved caspase 3	Cell Signaling	9664S	WB: 1:1000
Bcl-xl	Cell Signaling	2764S	WB: 1:1000
Bax	Cell Signaling	5023S	WB: 1:1000
Mcl-1	Cell Signaling	94296	WB: 1:1000
Bcl-2	Dako	M0882	WB: 1:1000
KRT19	Abcam	Ab195872	IF: 1:500
β-actin	Santa Cruz Biotechnology	sc-47778	WB: 1:5000
Ki-67	NOVUS	Nb500-170	IF: 4ug/ml

Abbreviations. IF: Immunofluorescence; WB: Western blot

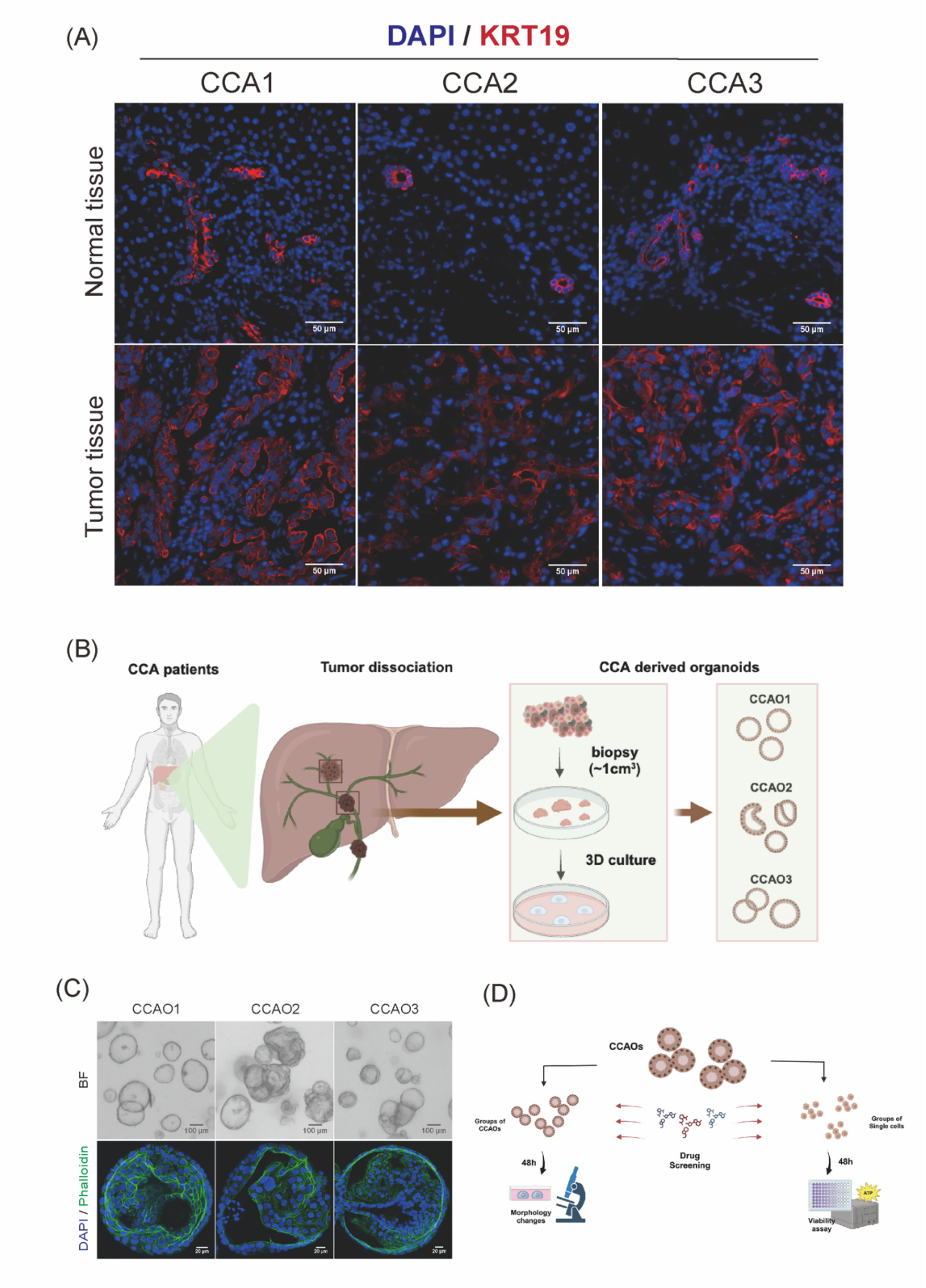

Figure S1. Cholangiocarcinoma tumor histology and sample processing. (A) Immunofluorescence staining (DAPI in blue, KRT19 in red) of tumor and adjacent tissue from three patients with CCA (CCA1, CCA2, and CCA3. (B) Schematic presentation of CCAOs initiation from human tumor biopsies collected during liver resection. (C) Representative brightfield and immunofluorescent images of three different CCAO lines. Nuclei are visualized by DAPI (blue) and actin filaments by phalloidin (green). (D) Schematic representation of the experimental set up in which CCAOs were exposed to different anti-tumor drugs for 48 hours. The effect of the drugs (gemcitabine 1µM, cisplatin 10µM, irinotecan 10µM, paclitaxel 1µM, and oxaliplatin 10µM) on cell viability was assessed by organoid morphology changes and CellTiter Glo assay, respectively.

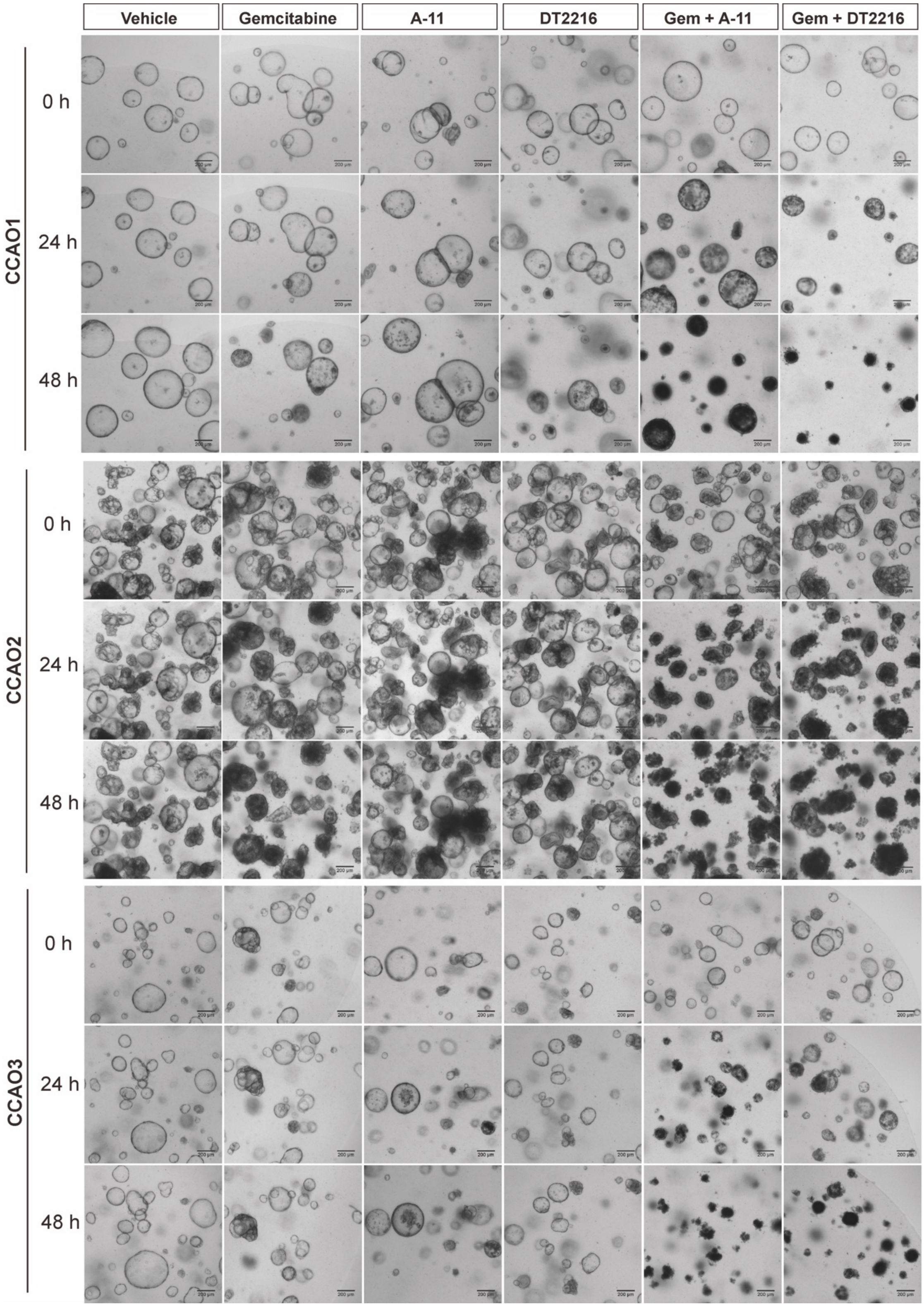

Figure S2. Brightfield images depicting morphological alterations in treated CCAOs were analysed at time points 0, 24, and 48 hours post-exposure to 1 μM gemcitabine, 5 μM A-11, 10 μM DT2216, or their combination.

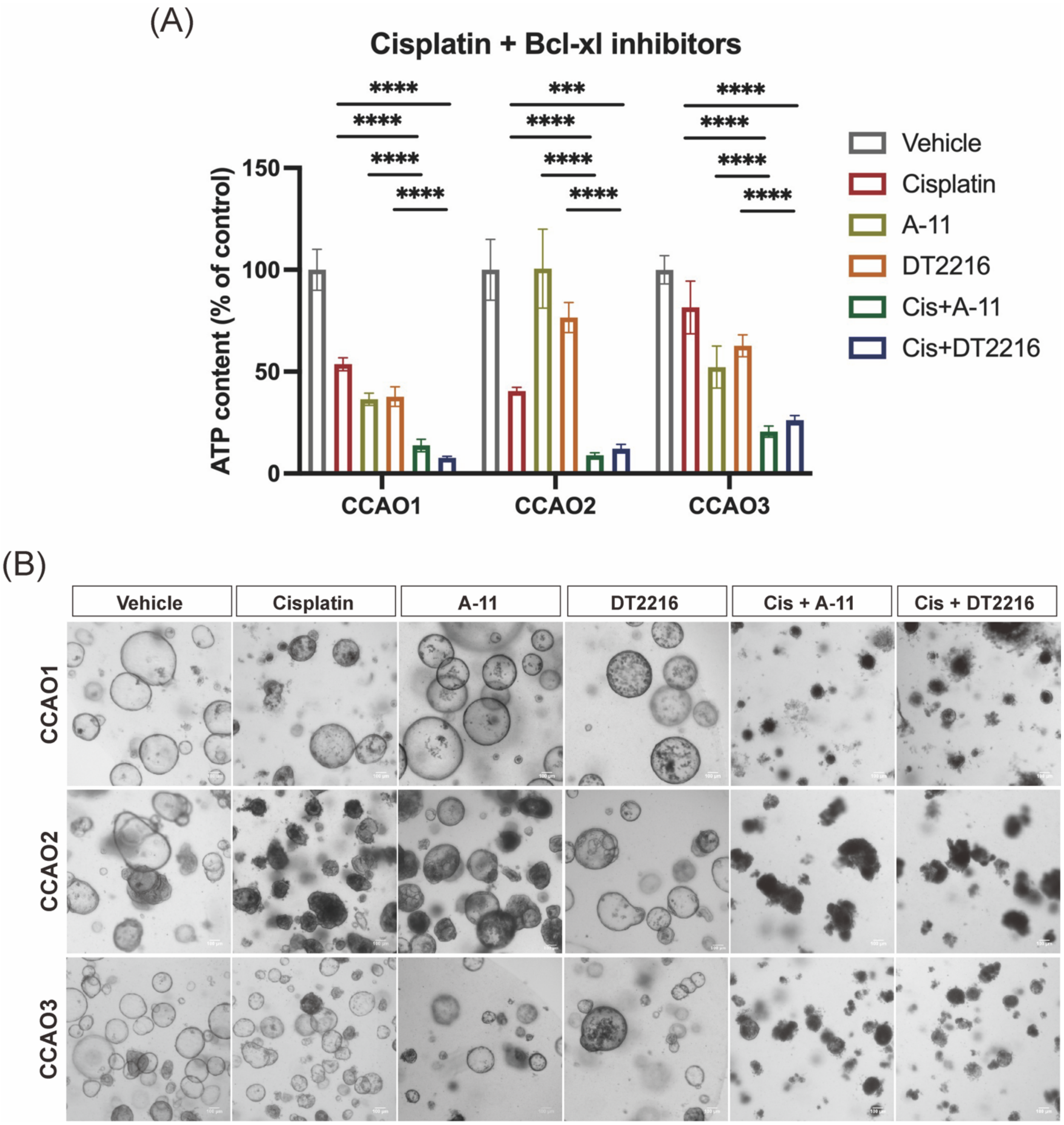

Figure S3. Drug responses to Cisplatin. A) Assessing cell viability by CellTiter Glo Assay post-treatment with 10 μM Cisplatin, 5 μM A-11, 10 μM DT2216, or their combination for 48h in the three CCAO lines. B) Following a 48-hour treatment, brightfield images were captured for CCAOs exposed to 10 μM Cisplatin, 5 μM A-11, 10 μM DT2216, or their combined treatment.
